# Does a physiotherapy programme of gross motor training influence motor function and activities of daily living in children presenting with developmental coordination disorder?

**DOI:** 10.4102/sajp.v72i1.304

**Published:** 2016-06-30

**Authors:** Sonill S. Maharaj, Riona Lallie

**Affiliations:** 1 Department of Physiotherapy, School of Health Sciences, University of KwaZulu-Natal, South Africa; 2 Physiotherapist, Durban North, South Africa

## Abstract

**Background:**

Children with developmental coordination disorder (DCD) lack motor coordination and have difficulty performing motor skills and activities of daily living. Research shows these children do not outgrow their motor difficulties and without intervention do not improve. Physiotherapy is relevant for these children, but due to limited clinical protocols for DCD the aim of this study was to determine the effect of a gross motor training programme for 6–12-year-old children with DCD.

**Methods:**

This randomised pre-test, post-test study recruited 64 children with scores of 15th percentile or below using the Movement Assessment Battery for Children (M-ABC). The children were divided equally into an intervention group receiving 8 weeks of gross motor training for core stability, strengthening exercises, balance and coordination with task-specific activities for 30 min per week, while the control group continued with general therapy and activities of daily living. The M-ABC and Developmental Coordination Disorder Questionnaire (DCDQ) were used to assess each child before and after 8 weeks.

**Results:**

Sixty children completed the study, with 43 males and 17 females (mean age 10.02 years, SD = 2.10). There were no adverse reactions to the programme and M-ABC scores for the intervention programme improved by 6.46%, ball skills (3.54%) and balance (4.80%) compared with the control (0.17%) and (0.15%), respectively. There were significant (*p* < 0.05) improvements in DCDQ scores, but teachers allocated lower scores than parents.

**Conclusion:**

This study supports 8 weeks of gross motor training which can be a beneficial intervention for physiotherapists to improve gross motor function for DCD.

## Introduction

Developmental coordination disorder (DCD) is characterised by impairments of motor coordination and affects an individual’s motor function, activities of daily living and academic achievements. The prevalence of DCD varies with the American Psychiatric Association (2000), indicating that approximately 5–6% of primary school children are affected. More recent estimates show that 1.7–3.2% of children present with DCD with a male to female ratio of 2:1 (Lingam *et al*. 2009; Pieters *et al*. 2011). DCD can persist into adolescence and adulthood, sometimes extending beyond the motor domain and including secondary mental health, emotional and behavioural issues (Zwicker *et al*. 2012).

The clinical presentation of DCD depends on the source of the disorder, severity, motor skills affected and environmental influences. Research has shown that children with DCD have deficits in gross and fine motor skills, postural control and proprioception with motor impairments manifesting in poor upper and lower limb movements (Missiuna *et al*. 2006; Summers, Larkin & Dewey 2008). Children with DCD can be differentiated by their motor and cognitive skills as they have poor static and dynamic balance, coordination, cognitive and general information processing (Asonitou *et al*. 2012). Historically, poor motor coordination in children was regarded as a developmental problem with terms such as ‘awkward’, ‘clumsy’, developmental apraxia, mild motor problems, low toned, perceptual motor difficulties, minimal brain dysfunction, minimal cerebral palsy or sensory integrative dysfunction used to describe these children (Dewey & Wilson 2001; Missiuna, Rivard & Bartlett 2003; Pearsall-Jones, Pik & Levy 2010).

Coordinated movements are regarded as mapping of perceptual (input) to motor (output) actions. This requires information processing at four sites which are sensation and perception, decision-making and planning, movement execution and feedback. A deficit in any one or more of these can result in poor motor co-ordination (Wilson & McKenzie 1998). The World Health Organisation introduced the International Classification of Functioning, Disability and Health (ICF 2001) which provides a framework for classification at three levels viz. body function and structures (impairment), activities (activity limitations) and participation (participation restrictions). This is based on the concept that impairments at the level of body function or structure influence the child’s ability to perform activities and participate in daily life and motor impairments leading to activity limitations and participation (Mandich, Polatajiko & Rodger 2003). Children with a diagnosis of DCD demonstrated greater amounts of muscular activity around the hip and shoulder musculature in comparison with children of similar ages (Johnston *et al*. 2002). They use ‘fixing’ of their joints during activities requiring stabilising one joint or part of the body so that another part can be moved with better control leading to stiff, awkward and clumsy movements and fatigue following their ‘fixing’ strategies (Missiuna *et al*. 2003). Additionally, children presenting with DCD activate their muscles during unilateral reaching movements by co-activation and delaying the onset of antagonist muscle activity with a longer duration of agonist activity. In asymmetrical bilateral reaching they change the onset of one or both agonist and antagonist muscle groups compared to normal developing children who change only the duration of antagonist muscle activity (Missiuna *et al*. 2003). A summary of clinical presentations of children with DCD is shown in Table 1.

**TABLE 1:** Clinical presentation of children with DCD.

Presentation of children with DCD	Authors
Completion of class work within normal time frame is challenging. They become distracted and frustrated with a straightforward task	Missiuna *et al*. (2003); Zwicker *et al*. (2012)
Joint laxity	Rivilis *et al*. (2011)
Short- and long-term memory impairments	Summers *et al*. (2008)
Poor sequencing, visual perception and spatial organisation	Summers *et al*. (2008)
Poor fine motor skills often affect dressing skills, for example, tying shoe laces and doing buttons independently	Summers *et al*. (2008)
Activities that require the coordination of both sides of the body is very complicated, for example, cutting with scissors, star jumps, and eating with a knife and fork	Dewey and Wilson (2001); Rodger *et al*. (2003); Summers *et al*. (2008)
Avoid socialising with peers. Some seek out younger children to play with while others go of on their own	Dewey and Wilson (2001); Missiuna *et al*. (2003)
Difficulty in organising his/her desk, locker and homework	Summers *et al*. (2008)
Avoid participation in gym class and the playground	Miller *et al*. (2001)
Low-frustration tolerance, poor self-esteem and lack of motivation	Dewey and Wilson (2001); Missiuna *et al*. (2003)

*Source*: Self compiled, Riona Lallie

Often children with DCD obtain poor grades at school, have poor core stability, endurance, difficulty with gross motor tasks such as throwing and catching a ball, learning and carrying out multiple and new tasks (Kolehmainen *et al*. 2011). This leads to non-participation in school activities and diminished motivation to interact socially. This results in the child having a low self-esteem and anxiety with a vicious cycle of spending less time with physical activities, developing inefficient motor and movement patterns and leading a sedentary lifestyle (Rivilis *et al*. 2011).

As a result of the complex clinical presentation of DCD there is uncertainty and controversy in the literature about the philosophies and intervention approaches for managing DCD. Studies have shown the importance of physical activity for health, growth and development for children with DCD and that without interventions to improve motor skills, physical and activities of daily living will not improve (Barnhart *et al*. 2003). A possible strategy to manage children with DCD to reach full movement potential and minimise coordination challenges and emotional and social problems is to vary the teaching strategies, content and therapy (Mandich *et al*. 2001). However, designing and implementing programmes for children with DCD is complex because of the heterogeneity of DCD and an intervention that is beneficial for some children may not be relevant or applicable for others (Dewey & Wilson 2001; Mandich *et al*. 2001).

The South African education authorities recognised the challenges associated with impairments, DCD and other neurological conditions in children who are unable to cope in mainstream schools. To cater for children with learning problems, physical disabilities or medical conditions special education schools were established with only *some schools* providing rehabilitation therapy, for example physiotherapy, audiology, speech and occupational therapy with psychological services. However, it was the researcher’s experience that due to resource and time constraints schools offering rehabilitation services sometimes considered children with minimal motor problems low priority and they did not get the benefit of these therapies. Understanding the complex nature of DCD and developing interventions to improve motor skills and functional activities of daily living will enable, inter alia, physiotherapists to manage children with DCD in small groups and avoid neglecting them due to various constraints (Dewey & Wilson 2001; Summers *et al*. 2008).

Therefore this study was designed to (1) determine the effect of an 8-weeks gross motor training programme using pre-and post-test scores of the Movement Assessment Battery for Children (M-ABC) and the Developmental Co-ordination Disorder Questionnaire (DCDQ) and (2) compare the allocation of DCDQ scores of parents and teachers following the programme for children presenting with DCD.

## Methodology

### Study design and sample selection

These randomised pre-test and post-test studies recruited children who were lagging behind their peers in motor proficiency at the Livingston Primary School in Durban. This is an English medium short-term remedial school for children experiencing learning difficulties such as dyslexia, dyspraxia, speech and language deficits, and hearing loss. The school has classes from Grades 1 to 7, providing holistic academic, therapeutic and cultural activities with children referred from mainstream schools within the Durban area. There is a social worker and there are rehabilitation services offering speech and audiology, occupational services and physiotherapy. After intensive interaction with the child for 2 to 3 years the child can return to mainstream schooling.

The children were identified by teachers in consultation with a senior occupational therapist as observation and clinical judgment are valid options to identify children with coordination difficulties (Miyahara & Wafer 2004). On identification, the children were included in this study if they had an M-ABC score of ≤ 15^th^ percentile, were in good general health and had the ability to comply with the gross motor training programme. Children having an IQ of less than 70, attending private physiotherapy or other physical activity programmes were excluded. Informed consent was obtained from teachers, parents and assents from the children to be assessed and participate in the study.

**FIGURE 1:**
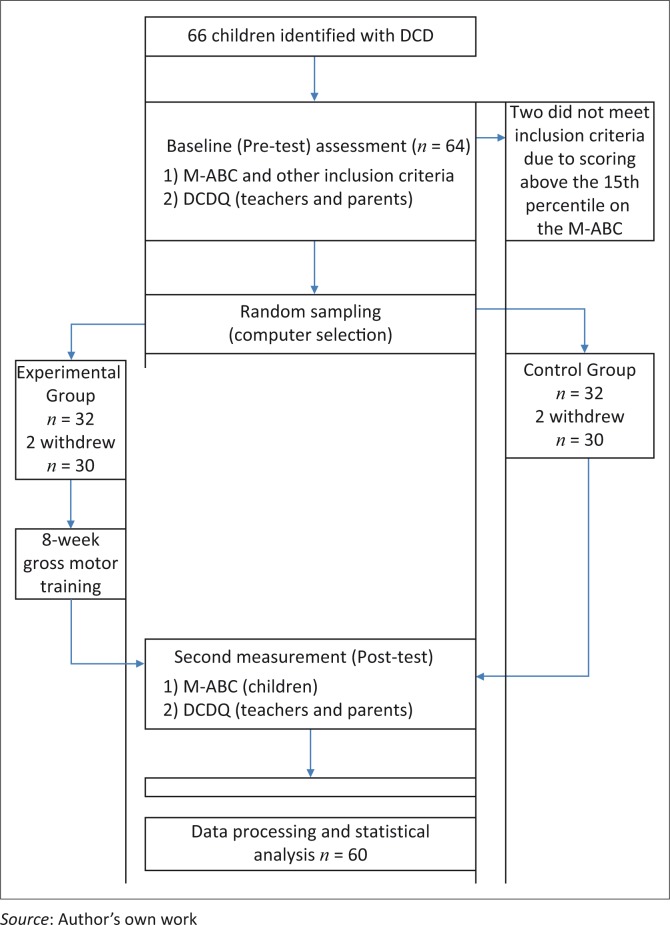
Flow diagram of study.

The M-ABC is considered the international gold standard for measuring motor coordination difficulties and has been purported to be a good indicator for the incidence of DCD (Dunford *et al*. 2004). The M-ABC is reliable, valid, responsive and precise, and is a standardised instrument to measure children with motor impairments and assess the efficacy of treatment programmes with a manual test–retest reliability score of 0.75 (Henderson & Sugden 1992). The DCDQ consists of 15 items with an overall sensitivity of age-specific cut-off scores exceeding 84% and specificity of 71% and correlates with M-ABC scores and is regarded as a valid screening tool for children ageing 5–15 years (Wilson *et al*. 2009). Sixty-six children between the ages of 6–12 years were identified with two children excluded because they scored above the 15th percentile. This resulted in a study sample of 64 children with the flow of the participants shown in Figure 1.

Ethical approval was obtained by the Biomedical Research Ethics Committee of the University of KwaZulu-Natal (BE287/12) and conformed to the Helsinki Declaration standards (WMA 2013). Permission to use the school was granted by the principal and KwaZulu-Natal Department of Education. The study was conducted from February to November 2013 and participation in the study was voluntary, with the child allowed to withdraw at any point. Confidentiality was maintained by coding all data and files, storing them in a computer and securing them by a secret password accessed only by the researchers.

### Procedures

A draft gross motor training programme was developed by reviewing the programme of play and group therapy used by the therapists at the school. Based on a literature review of exercise programmes for children with impairments, feedback from clinicians and what senior therapists experienced in neuro-developmental therapy, the draft programme was reviewed for strengthening exercises, core stability, balance and coordination. This was based on the researcher’s view that best practices emerge from published evidence, expert opinion and the patient’s needs and preferences (Sackett *et al*. 1996).

The programme incorporated throw, catch and ball activities with target throwing which focused on strength and coordination which mimic sensory integration exercises that facilitate and enhance motor skill development in children with DCD (Sugden 2007; Tsai *et al*. 2009). The draft programme was used in a pilot study on children with DCD attending a private facility, and after feedback from parents, teachers and therapists a final programme was designed. This was an 8-week gross motor training programme for core stability, strengthening exercises, balance and coordination with task-specific activities for 30 min per week to improve ball skills, balance, bilateral hand function, postural control and core stability (Appendix A). A school term consists of approximately 10 weeks which was sufficient for the eight weeks of gross motor training and pre- and post-testing.

These 64 children were divided equally into an intervention or control group using simple randomisation by means of the *Clinstat* computer programme which was administered by the school secretary who allocated the groups and was independent of the study. The control group continued with general therapy and activities of daily living in their classrooms during which children in the gross motor training programme performed their activities in small groups of seven or eight in the Occupational Therapy Department which was set to specifications as stipulated in the M-ABC manual (Henderson & Sugden 1992). Based on ethical requirements, on completion of the study children in the control group crossed over and received the gross motor training programme. The procedure for testing and scoring of the M-ABC test is shown in Table 2. All pre-tests’ and post-tests’ scores were recorded by two research assistants who were experienced with the instrument and independent of the study. Pre- and post-DCDQ scores were recorded by teachers and parents. As a safety precaution, a first-aid kit was available for any minor injuries, and a nurse and doctor were ‘on call’ in the event of any adverse reactions during the activities.

**TABLE 2:** Domains of Movement Assessment Battery for Children (M-ABC) tested with relevant age groups.

Ages	4, 5 and 6 years	7–8 years	9–10 years	11–12 years
Manual dexterity	Posting coins, threading beads, bicycle trail	Placing pegs, threading lace, flower trail	Shifting pegs, threading nuts on a bolt, flower trail	Turning pegs, cutting-out elephant, flower trail
Ball skills	Catching a bean bag, rolling ball into a goal	One hand bounce and catch, throwing bean bag into a box	Two-hand catch, throwing bean bag into the box	One hand catch, throwing at wall target
Balance	One-leg standing (static), jumping over the cord (dynamic), walking heels raised (dynamic)	Stork balance (static), jumping in squares (dynamic), heel-to-toe walking (dynamic)	One-board balance (static), hopping in squares (dynamic), ball balance (dynamic)	Two-board balance (static), jumping and clapping (dynamic), walking backwards (dynamic)

*Source*: Self compiled, Riona Lallie

### Data analysis

Analysis of data was performed by means of the SAS software version 9.3 (SAS Institute Inc., Cary). Normality of data was assessed using the Shapiro–Wilk test with data normally distributed, and an intention to treat (ITT) analysis was done by means of imputation of the mean of the other group (MOTH). Means and standard deviations were used to show M-ABC and DCDQ scores with ball and balance skills, measured before and after intervention. An unpaired *t*-test and analysis of variance (ANOVA) were used to compare the control and intervention groups by comparing the scores of ball and balance skills to determine the impact of the intervention. Results are depicted by means of tables and graphs using Microsoft Excel 2010. Statistical significance was noted if *p* < 0.05.

## Results

Sixty-four children satisfied the inclusion criteria, but two from the control and one from the gross motor training programme withdrew for personal reasons. A child from the gross motor training programme was transferred to another school, resulting in 60 children completing the study for data analysis. The flow of the participants is shown in Figure 1. There were 43 boys and 17 girls with mean age of 10.02 years (SD = 2.10). The mean age of children in the gross motor training programme was 10.11 (SD = 1.98) and the control 9.9 (SD = 2.44) years with no significant difference between groups (*p* = 0.893). The M-ABC assessment ranged between the 1st percentile and the 15th percentile, and the post-intervention scores ranged from the 1st percentile to the 32nd percentile with the scores categorised as category 1 (below 5th percentile); category 2 (from the 5th percentile to the 15th percentile) and category 3 (above the 15th percentile). There were no deviations from the protocol or adverse reactions during and after the gross motor training programme.

There were significant (*p* = 0.031) improvements post-testing for M-ABC scores with a mean increase of 6.46% compared to children in the control who increased their scores by 0.33%. From the 14 children in category 1, six moved to category 2; and from the 16 children in category 2, ten moved into category 3 and were considered ‘normal’, no longer requiring physiotherapy. The control group had 6 children in category 1 and 24 in category 2 with no significant changes in any of their scores (Table 3). M-ABC scores for ball skills and balance improved by an average of 3.54% and 4.80%, respectively, in the gross motor programme group compared with the control group, improving by an average of 0.17% and 0.15%, respectively. The DCDQ scores for children in the gross motor training programme also improved significantly with no significant improvements for those in the control group. It was noted that although parents and teachers showed higher DCDQ scores, there were no significant differences between their scores (*p* = 0.069) after the gross motor training programme. However, teachers allocated lower scores for the child compared with the parents with five teachers and twelve parents scoring the child into a higher category. The significant improvements within the intervention group in ball skills (*p* = 0.021), balance (*p* = 0.033) and DCDQ scores of parents (*p* = 0.038) and teachers (*p* = 0.044) and between group significance for the intervention with M-ABC (*p* = 0.041); ball skills (*p* = 0.048); balance (*p* = 0.034) and DCDQ for parent (*p* = 0.024) and teachers (*p* = 0.045) are shown in Tables 3, 4 and 5 respectively.

**TABLE 3:** Movement Assessment Battery for Children (M-ABC) scores pre- and post-intervention.

M-ABC	Pre-test % (Mean SD)	Pre-test % (Mean SD)	Mean Post-Pre- difference % (SD)	*p*-value	Between group comparison Z ES (*p*-value)
Intervention group	7.61 (4.32)	14.07 (8.02)	6.46 (4.56)	0.031	
Control group	8.15 (4.40)	8.48 (4.31)	0.33 (0.55)	0.69	*p* = 0.041*
				0.770	

*Source*: This is the statistical value following the study

* *p* < 0.05 indicates statistical significance; Z ES denotes effect size.

**TABLE 4:** Scores of ball skills and balance pre-and post-intervention.

Variable	Pre-test % (Mean SD)	Post-test % (Mean SD)	Mean difference % (SD) (improvement)	*p*-value	Between group comparison Z ES (*p*-value)
**Ball skills**					
Gross motor training	7.06 (2.18)	3.52 (2.34)	3.54 (1.02)	0.021*	0.84(0.048)*
Control	6.69 (2.10)	6.52 (2.15)	0.17 (0.50)	0.990	-
**Balance**					
Gross motor training	9.66 (2.76)	4.86 (2.39)	4.80 (1.22)	0.033*	1.17(0.034)*
Control group	10.17 (2.89)	10.02 (2.87)	0.15 (0.36)	≤ 0.819	-

*Source*: This is the statistical value following the study

* *p* < 0.05 indicates statistical significance; Z ES denotes effect size.

**TABLE 5:** DCDQ scores pre-and post-intervention.

Group	Pre-test % (Mean SD)	Post-test % (Mean SD)	Mean Post-Pre difference % (SD)	*p*-value	Between group comparisons Z ES (*p*-value)
**Parents**					
Intervention group	36.07 (5.92)	45.86 (8.23)	9.79 (4.86)	0.038*	0.51(0.024)*
Control group	35.96 (6.14)	36.11 (6.07)	0.15 (0.77)	0.910	
**Teachers**					
Intervention group	30.00 (6.72)	48.79 (10.75)	18.79 (5.86)	0.044*	1.82(0.045)*
Control group	30.04 (6.72)	30.93 (7.10)	0.89 (1.40)	0.960	

*Source*: This is the statistical value following the study

* *p* < 0.05 indicates statistical significance; Z ES denotes effect size.

## Discussion

Studies of children presenting with DCD show that boys are more affected than girls, but when engaging in physical activities they overcome their poor movement patterns and impairments by improving their motor skills, muscle strength, endurance and activities of daily living (Barnhart *et al*. 2003; Pieters *et al*. 2011; Riethmuller, Jones & Okely 2009). The results of this study support these studies as more boys than girls were affected and following the gross motor training programme of exercises there were significant improvements in M-ABC and DCDQ scores. However, this study is contrary to the study by Pless and Carlsson (2000) who indicated that there were no significant differences in M-ABC scores after an exercise programme for DCD children having borderline motor problems and recommended that the children would require more specific therapy. This study supports exercises used in physiotherapy as being relevant and specific, as the researchers were physiotherapists and facilitated the exercise programme ensuring that each movement pattern was emphasised, repeated and adhered to basic movement patterns for stability and coordination which is required to improve the activity (Wilson 2005). The programme also incorporated star jumps, skipping, throwing and catching to improve inter-limb and eye–hand coordination with physical, visual and verbal prompts, which may have improved motor scores. It is noted that children with DCD have difficulty with visual-spatial processing and sensory integration. This affects perception with eye–hand and inter-limb coordination because of poor proprioception, motor sequencing and timing, which affects activities like catching a ball, running, climbing and intercepting objects (Cantell, Smyth & Ahonen 2003; Missuina, Rivard & Bartlett 2003; Zoia *et al*. 2006). It is possible that the gross motor training used in this study requiring throw and catch activities, target throwing and ball activities focused on strength and coordination which facilitated and enhanced motor skills by mimicking sensory integration in these children with DCD as postulated by Sugden (2007) and Tsai *et al*. (2009).

There is also evidence that maturation did not improve balance in children with DCD, but that balance activities in a structured environment were necessary (Fong, Tsang & Ngo 2012). The improvement in balance following the gross motor programme may be related to balance being either ‘static’, requiring maintaining different postures, or ‘dynamic’, requiring activities while moving. This could have been facilitated by the child engaging in the programme requiring perception of the centre of gravity with timely motor response to realign the centre of gravity. This would be possible by the perceptual motor activity that involves the sensory and motor systems, which are activated by the sensory system receiving information from the environment and forwarding this information to the central nervous system for a reaction (Cherng, Hsu & Chen 2007).

A study on a strengthening programme for a child with DCD found that muscle strength, body awareness and proprioception improved postural muscle activity and proximal stability. This is due to children with DCD having a proximal to distal muscle activation sequence contributing to poor upper limb coordination (Cherng *et al*. 2007; Geuze 2005; Kaufman & Schilling 2007). The improvements noted in this study may relate to the child’s improved postural control and core stability that provided a foundation for greater force production in the upper and lower extremities which could have been transferred to other skills and activities of daily living contributing to improved DCDQ scores.

Participation in activities is important for a child’s development. This enables them to attain the social and physical competencies required to provide social and emotional harmony, sense of meaning and purpose in life with successful outcomes motivating the child to try new challenges (Kolehmainen *et al*. 2011). It is possible that by participating in the gross motor programme with purposeful and enjoyable play activities, the children were motivated to interact with their peers. This is supported by anecdotal reports from parents and teachers who indicated that they observed secondary emotional and behavioural changes as the children enjoyed working in groups and cooperated in the activities. They also seem to have improved their listening and attention skills by complying with instructions and waiting patiently for their turn to share equipment.

Although this study found significant improvements in DCDQ scores, some teachers scored the children much lower than their parents. The researchers presume that this may relate to teachers having more knowledge of children with DCD and spend more time with the children in a structured environment, where they experience the children coping with motor functions in the classroom and the playground (McDavid, Cox & Amorose 2012). This is supported by Wilson *et al*. (2012) that a lower percentage of parents were aware of coping strategies compared with teachers.

## Conclusion

This study supports an 8-week gross motor training programme for use by physiotherapists as a potential exercise intervention for 6–12-year-old children with DCD because the results improved M-ABC and DCDQ scores. Anecdotal reports also indicate that the children enjoyed the programme and that their participation improved their social interactions. Currently, the number of children attending remedial schools is increasing with the ratio of therapists to manage them posing a challenge. The use of the 8-week gross motor training programme and small group interactions will therefore be a beneficial exercise intervention for physiotherapists to improve the management and activities of daily living in children with DCD. However, further research is required to determine whether the improvements obtained will be evident later in their lives or be sufficient to meet participation levels for activities like sports and games that require more complex information processing.

Some of the limitations of this study were the time constraints of the academic term of 10 weeks. Additionally, standardised tests, such as M-ABC, do not measure the quality of movement, motor-planning problems and psychosocial consequences, such as self-esteem and confidence, which are functional problems often reported in children with DCD. Other limitations of this study were: it was a single-centred study where therapists and teachers could have been biased as they were in daily contact with the children, there was no attempt to stratify by age and the carry-over effect of the intervention was not determined. As the age range of the children in this study is fairly broad, further research is warranted to stratify children by age to determine if this programme can serve as an early intervention approach. A further study of the carry-over effect to determine how often the programme should be repeated to ensure optimal maintenance of function would also be useful.

## APPENDIX A

### Week 1

Warm up

1. Jogging on the spot (1 minute)
2. Stride jump – Child flexes alternate knees and hips to touch arms on the side (1 minute)
3. Stride Jump – Hands on shoulders and child touches right elbow to left knee, then left elbow to right knee (1 minute)

[Each warm-up exercise is done for half a minute for 6–8 year olds]

1. Child lies supine on a wedge. Therapist throws a ball at them. The child catches a ball, sits up and throws it back at the therapist. (Repeat 10 times).
2. Sit-ups to the side. Child lies crook lying on the wedge, hands on shoulders. Child lifts up with right elbow touching left knee and then left elbow to right knee. (Hold to the count of 5) (Repeat 5 times on each side).
3. Push-ups. Child starts in 4 pt kneeling on the floor with their feet crossed together. Push down on their arms. (Repeat 10 times).
4. Sitting on big ball with hips and knees at right angles. Child sits upright and bounces on the ball while being encouraged to maintain abdominal contractions (Repeat for 2 minutes).
5. Star jumps in a hula-hoop. Child starts with feet together in the middle of the hula-hoop. They then jump legs apart and arms abducted at the same time. (Repeat 30 times).
6. The hoop is placed on the floor and the child jumps in and out of it. This is done in all directions – right, left, backward and forward (10 times in each direction).
7. Child is to balance on 1 leg on a small ball and throw beanbags through the hoop (Repeat 10 times on each leg).
8. Five hoops on the floor in front of each other. Child has to hop on 1 leg in each hoop and back. Both legs are done.
9. Two children stand on the wobble boards. Catches a bean bag and throws it back to each other.

Cool down: deep breathing exercises and stretches

### Week 2

Warm up

1. Tummy crunches. Child lifts head and shoulders of the mat and reaches forward. Hold for 5 seconds (Repeat 5 times).
2. Sit-ups using a wedge, as well as sit- ups to the sides using the wedge (Repeat 5 times forward and to each side).
3. Push-ups. Child lies with tummy on a block. Arms out in front, flexed and internally rotated on floor. Pushes down on arms (Repeat 10 times).
4. Child sits on a ball. Throws a bean bag up in the air and catches it. Abdominal contractions are encouraged (Repeat 10 times).
5. Child sitting on a medium-size ball. Bean bags are placed on each side of the ball, about half a meter away. Child reaches for bean bag, while maintain balance on the ball and then throws it into a hoop, placed 2 meters in front of child (Repeat 10 times on each side).
6. Star jumps. Done without hula-hoop (Repeat 30 times).
7. Child jumps in and out of the hoop. In all directions – right, left, backward and forward. First in a specific direction and then a combination of directions.
8. Children get into pairs. Throw a 20-cm ball to each other. (Underarm and overarm) (Repeat 10 times each).
9. Child jumps on the trampoline. Every alternate jump is a star jump (Repeat 10 times).
10. Two children stand on wobble boards in front of each other and throw tennis balls to each other.

Cool down: deep breathing exercises and stretches.

### Week 3

Warm-up

1. Tummy crunches in crook lying. Lift head and right shoulder of the mat and touch left knee. Hold for 7 seconds (Repeat 5 times on each side).
2. Child in supine but resting on forearms so head and shoulders are of the mat. Bicycle legs are done (Repeat for 2 minutes).
3. Sit-ups (forward and to each side), using less of the wedge.
4. Push-ups. Lie prone on a medium-size ball so that they are weight bearing with arms on the mats and their thighs are resting on the ball. Push through arms.
5. 4 point kneeling. Child lifts right arm and left leg of the mat. Hold for 7 seconds (Repeat 10 times on each side.
6. Stride and scissor jumps (Alternate) (Repeat 10 times for each jump).
7. Sitting on the ball. Throw a bean bag in the air, clap and catch the bean bag (Repeat 10 times).
8. Child bounces a tennis ball on the floor and catches it.
9. Child uses the hula-hoop to skip, using both feet to jump (Repeat 10 times).
10. Child balances on each leg for 20 seconds. Child is not allowed to hook their leg around the other.

Cool down: deep breathing exercises and stretches.

### Week 4

Warm-up

1. Tummy crunches. (As per week 3 but to hold for 10 seconds).
2. Sit-ups. Done forward and to each side, not using the wedge. (Repeat 10 times. Sit-ups will be increased 5 times forward and to each side every week).
3. Child to sit on ball. They will slide down the ball into supine, keeping knees and hips at 90 degrees, without using their hands. Then sit up (Repeat 10 times).
4. Races. Children do bear walking for 5 meters forward and 5 meters backwards.
5. Use the hula-hoop around the waist. Continue for 1 minute (Repeat 3 times).
6. Throw a tennis ball at a target on the wall and catch it (Repeat for 3 minutes).
7. Star jumps (Repeat 40 times).
8. Children get into pairs, standing in front of each other. They throw a medium-sized ball from above their heads to the other child. The ball must bounce in the centre (Repeat 20 times).
9. Children to stand on wobble boards in front of each other, throw a tennis ball to each other, which must bounce in the centre.
10. Tandem walking along a 5 meter line (6–8 year olds to walk forward and 9–12 year olds to walk backwards).

Cool down: deep breathing exercises and stretches.

### Week 5

Warm up

1. Tummy crunches. Lift head and shoulders of the mat and reaching forward, at the same time flex hips to about 20 degrees. Hold for 5 seconds (Repeat 5 times).
2. Sit-ups (Repeat 15 times).
3. Child lies supine but resting on forearm so head and shoulders are of the mat. Child does scissor legs and legs up/ down without touching the mat (Repeat 10 times each leg exercise).
4. Child gets into pairs and lies in prone facing each other with head and chest of the mat. They roll the ball to each other (Repeat 20 times).
5. Sitting on a big ball. Child juggles 2 bean bags while contracting abdominals (Repeat for 2 minutes).
6. Child stands with a medium-sized ball against their back against a wall. Does squats and holds for 20 seconds (Repeat 10 times).
7. Skipping with the hoop. To jump with both feet. (Repeat 20 times).
8. Child bounces a tennis ball on the floor and catches it with one hand. Do both sides. (Repeat 10 times).
9. Children to get into pairs and a throw a Frisbee to each other and catch it (Repeat 15 times).
10. Child to balance with 1 leg on a small ball and to juggle 2 bean bags (Repeat for 2 minutes).

Cool down: deep breathing exercises and stretching.

### Week 6

Warm-up

1. Tummy crunches (As per week 5 but to hold for 7 seconds).
2. Sit-ups (Repeat 15 times).
3. Push-ups. Child lies prone on hands and toes, pushes through arms (Repeat 10 times).
4. Child in standing but bends over to put hands on a block in front of him and pushes on hands and jumps over to block to each side (Repeat 10 times to each side).
5. Races. Spider walking and bear walking for 5 meters, forward and backward.
6. Star jumps (Repeat 50 times).
7. Hula-hoop around waist and arm (Repeat for 2 minutes on the waist and for each arm).
8. Skipping, now with skipping rope (Repeat for 2 minutes).
9. Bounce tennis balls on the floor and catch it. Use alternate hands to do this (Repeat for 1 minute).
10. Child to balance with 1 leg on a trampoline and throw a bean bag in a hoop placed 3 meters away (Repeat 10 times on each leg).

Cool down: deep breathing exercises and stretches.

### Week 7

Warm-up

1. Tummy crunches continued.
2. Sit-ups.
3. 4 point kneeling. Lift right arm and left leg of the mat. Hold for 15 seconds (Repeat for 10 times on each side).
4. Child lies prone on a ball and walks forward until the ball is under their knees. The therapist rolls a big ball to each child, the child rolls the ball back using one hand (Repeat 10 times on each side).
5. Sit on a big ball. Without using hands, pick 1 leg up and hold for 10 seconds (Repeat 5 times on each side).
6. Star jumps (repeat 50 times).
7. Skipping (As per week 6).
8. Eight hoops placed in front of each other. Child to hop on 1 leg into the hoop, then jump into the next and continue (Repeat twice).
9. Children to get in pairs. Balance on 1 leg and throw the Frisbee to the other child.

Cool down: deep breathing exercises and stretches.

### Week 8

Warm-up

1. Sit-ups.
2. Push-ups.
3. Children get into pairs. Lie prone with arms and chest, as well as legs of mat. Hold for 20 seconds. (Repeat 15 times).
4. Children get into pairs to do wheel barrow races. They have to go with a bean bag, walk forward and throw into a bucket. Each child will have a turn (Repeat 5 times).
5. Lie in supine but resting on forearms. Heavy ball is placed between ankles. Child has to lift the ball to 50 degrees and hold for 10 seconds (Repeat 10 times).
6. Skipping (Repeat 15 times).
7. Star jumps (Repeat 50 times).
8. Sitting on big ball. Lift 1 leg of and touch knee with opposite hand. Hold for 10 seconds (repeat 10 times on each side).
9. Get into pairs. Two tennis balls are used for a pair. Each must throw and catch at the same time. (Repeat 1 minute).
